# Detection and Analysis of Conveyor Belt Damage: A Review of Sensing Technologies and Signal-Based Approaches

**DOI:** 10.3390/s26144453

**Published:** 2026-07-13

**Authors:** Aleksandra Rzeszowska, Ryszard Błażej, Leszek Jurdziak

**Affiliations:** Faculty of Geoengineering, Mining and Geology, Wrocław University of Science and Technology, na Grobli 15 St., 50-421 Wrocław, Poland; aleksandra.rzeszowska@pwr.edu.pl (A.R.); leszek.jurdziak@pwr.edu.pl (L.J.)

**Keywords:** conveyor belt diagnostics, fault diagnosis, condition monitoring, non-destructive testing, sensing technologies, signal processing, feature extraction, machine vision, magnetic flux leakage, multimodal data fusion

## Abstract

**Highlights:**

**What are the main findings?**

- Conveyor belt diagnostics increasingly combine sensing technologies, signal processing, and artificial intelligence to improve damage detection capabilities.
- Existing diagnostic approaches remain predominantly sensor-specific, creating challenges related to multimodal data integration and result comparability.

**What are the implications of the main findings?**

- Development of unified damage representation frameworks may improve transferability and scalability of future diagnostic systems.
- Integration of heterogeneous sensing technologies with intelligent data processing represents a promising direction toward predictive and autonomous conveyor belt monitoring.

**Abstract:**

Conveyor belts constitute critical components of bulk material handling systems, and their reliable operation directly affects process continuity, operational safety, and maintenance costs in industrial environments. Increasing requirements regarding system reliability and predictive maintenance have stimulated the development of advanced diagnostic methods for conveyor belt condition monitoring. This review presents a comprehensive analysis of conveyor belt damage detection and diagnostic approaches, with particular emphasis on sensing technologies and signal-based methodologies. The paper discusses major conveyor belt degradation mechanisms and analyzes their representation in diagnostic data obtained using different sensing modalities. Current developments in machine vision systems, magnetic methods based on magnetic flux leakage, ultrasonic techniques, and X-ray imaging are critically reviewed together with signal preprocessing procedures, feature extraction strategies, and damage classification approaches. Particular attention is devoted to the transition from conventional signal processing techniques toward machine learning and deep learning methods enabling automated feature representation and fault identification. The analysis indicates that despite substantial progress in sensing technologies and artificial intelligence, most existing solutions remain strongly sensor-specific and limited to individual data modalities. Key research gaps include the lack of unified damage representation frameworks, limited benchmark datasets, and the insufficient integration of multimodal sensing information. Future progress will likely depend on the development of integrated diagnostic ecosystems combining heterogeneous sensing technologies, advanced feature representation methods, and intelligent decision-support systems.

## 1. Introduction

Conveyor belts are among the most important components of bulk material handling systems and are widely used in mining, power generation, cement production, and industrial logistics, wherever continuous transportation over medium and long distances is required [1,2,3]. Their widespread application results from their high transport capacity, ability to operate continuously, and relatively low operational costs compared to alternative material transport systems [4,5,6]. However, conveyor belt systems are simultaneously exposed to intensive operational loads and harsh environmental conditions, which lead to the gradual degradation of their technical condition.

The reliability of conveyor belts is crucial for maintaining the continuity of industrial processes. Belt failures may result in unplanned downtime and significant economic losses, particularly in large-scale industrial facilities such as open-pit mines and mineral processing plants [7,8]. Moreover, conveyor belt failures may create safety hazards for workers and negatively affect the environment, especially during the transportation of hazardous or dusty materials. Consequently, conveyor belt diagnostics have become an essential element of maintenance strategies and industrial infrastructure monitoring systems [9,10,11,12].

The degradation of conveyor belts is a complex phenomenon resulting from the interaction of multiple mechanical, operational, and environmental factors. The most significant damage mechanisms include abrasive wear caused by contact with transported material, impact damage associated with material drop, fatigue degradation resulting from cyclic loading, and damage caused by improper belt tracking [13]. These mechanisms lead to various forms of degradation, including surface wear, cuts and longitudinal rips, perforations, delamination, and steel cord defects in steel-cord conveyor belts [13]. The diversity and often irregular nature of these defects make their reliable identification a major diagnostic challenge.

In response to the increasing demand for reliable conveyor belt operation, numerous sensing technologies have been developed over recent decades to monitor belt condition. The most commonly used approaches include machine vision systems, magnetic methods, ultrasonic techniques, and X-ray inspection systems [14,15]. Vision-based systems employing industrial cameras and advanced image-processing algorithms enable real-time detection of surface defects and are widely applied in industrial environments [16,17,18]. Magnetic methods, particularly those based on magnetic flux leakage analysis, allow the assessment of steel cord condition and the detection of internal defects invisible on the belt surface [15,19,20,21]. Ultrasonic techniques are primarily used for belt thickness measurement and wear evaluation, providing information about the degree of material degradation [15,22,23,24]. In contrast, X-ray systems enable the detailed inspection of the internal belt structure; however, their industrial application is often limited by high implementation costs and operational requirements [14,25,26,27].

Despite significant advances in sensing technologies, the interpretation of diagnostic data remains a major challenge. Signals generated by different measurement systems differ considerably in their physical nature, structure, and dimensionality. Image data, one-dimensional signals, and thickness profiles require different analysis approaches, leading to substantial diversity in the methods reported in the literature [9,28,29]. Numerous signal-processing and feature-extraction techniques have therefore been developed, including classical statistical measures as well as advanced methods based on frequency analysis, wavelet transforms, time–frequency analysis, and machine learning approaches [20,28,29,30].

In recent years, increasing attention has also been devoted to the application of artificial intelligence methods, particularly deep learning, for the automatic detection and classification of conveyor belt defects [18,31,32,33,34,35]. These approaches can achieve high detection accuracy; however, they usually require large training datasets and often suffer from limited interpretability. Furthermore, most proposed methods are strongly associated with specific input data types, which restricts their applicability across different sensing systems.

Consequently, one of the major challenges in conveyor belt diagnostics remains the lack of a unified approach to damage representation and diagnostic feature extraction. Existing methods are typically designed for specific sensing technologies and do not provide a consistent interpretation framework. This hinders both the comparison of diagnostic performance across different methods and the development of universal damage classification algorithms.

Several review papers addressing conveyor belt diagnostics have been published in recent years. Recent reviews have primarily focused on non-destructive testing technologies and sensing hardware for conveyor belt inspection [14,15]. While these studies provide valuable overviews of available diagnostic techniques, they mainly discuss sensing principles, measurement systems, and defect detection capabilities. Comparatively less attention has been devoted to the relationship between physical damage mechanisms, their representation in diagnostic data, and the signal-processing and artificial intelligence methods used for damage interpretation. As a result, the literature remains fragmented, with individual sensing modalities typically analyzed separately and without a common framework enabling the comparison of diagnostic information generated by different measurement systems.

Therefore, there is a need for a review that moves beyond the description of sensing technologies and focuses on the transformation of physical damage into diagnostic information. Such a perspective enables the comparison of different sensing modalities at the level of signal representation, feature extraction, and intelligent interpretation methods. It also creates an opportunity to identify common challenges related to data fusion, transferability of diagnostic models, and the development of unified damage representation frameworks.

The aim of this paper is to provide a critical review of conveyor belt damage detection and analysis methods, with particular emphasis on the relationship between sensing technologies, signal representation, feature extraction, and artificial intelligence-based diagnostic approaches. Unlike previous reviews focused primarily on sensing hardware and non-destructive testing techniques, this work analyzes how different damage mechanisms are transformed into diagnostic data and how these representations influence subsequent processing, classification, and decision-making stages. Furthermore, the paper identifies current limitations related to data representation, multimodal integration, and diagnostic model transferability, and discusses future directions toward unified and more generalizable conveyor belt monitoring frameworks.

## 2. Methodology of the Review

This paper presents a structured narrative review prepared using a transparent literature search and selection strategy. The methodological section was developed with reference to the general principles of transparent literature review reporting and following selected PRISMA 2020 recommendations adapted for a structured narrative review; however, due to the narrative character of the study, no formal systematic review or meta-analysis was conducted [36].

The literature search was performed using the Scopus, Web of Science, and Google Scholar databases, as well as publisher resources including ScienceDirect, SpringerLink, IEEE Xplore, and MDPI. Additionally, the Publish or Perish software (version 8.19.5300; Tarma Software Research Ltd., London, UK) was used to compare search results obtained for different keyword combinations, identify highly cited publications, and support the preliminary assessment of source relevance. The search strategy was based on combinations of keywords related to conveyor belt diagnostics, sensing technologies, signal analysis, and damage classification using logical operators such as AND and OR. The primary search was conducted using the query:

TITLE-ABS-KEY (“conveyor belt” AND (damage OR defect OR fault) AND (diagnostics OR detection OR monitoring))

in the Scopus database and the equivalent query

“conveyor belt” AND (damage OR defect OR fault) AND (diagnostics OR detection OR monitoring)

using Google Scholar through the Publish or Perish software.

**Figure 1:**
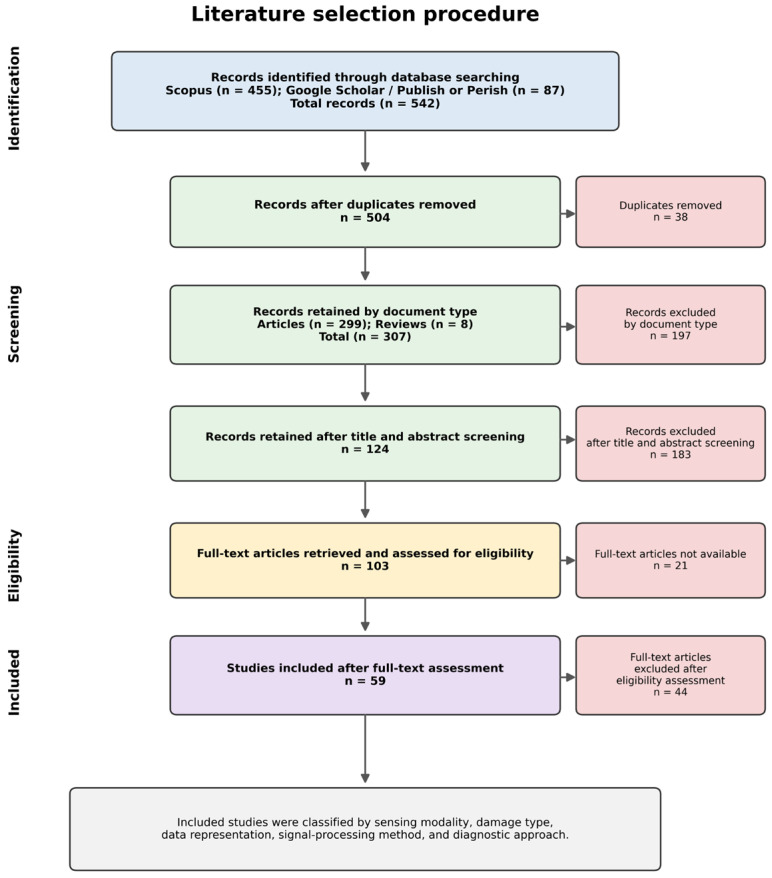
PRISMA-inspired flow diagram of the literature identification, screening, eligibility assessment, and study inclusion process used for the qualitative synthesis of conveyor belt diagnostic methods.

The initial search identified 542 records, including 455 records from Scopus and 87 records from Google Scholar. After duplicate removal, 504 records remained for further screening. The literature selection procedure is summarized in Figure 1.

The review primarily included peer-reviewed English-language publications directly related to conveyor belt damage, defect detection methods, and diagnostic data processing. Selected studies from related research areas, including condition monitoring, nondestructive testing, and signal analysis, were also considered when they provided relevant methodological contributions to the investigated problem. Preference was given to publications in recognized scientific journals, recent studies, and highly cited papers. The inclusion criteria comprised peer-reviewed journal articles and review papers written in English and directly related to conveyor belt damage, condition monitoring, sensing technologies, signal processing, feature extraction, or diagnostic algorithms. Conference proceedings, patents, non-English publications, and studies not directly related to conveyor belt diagnostics were excluded from further analysis.

The selected publications were analyzed with respect to the investigated damage types, applied sensing technologies, input data characteristics, signal-processing and feature-extraction methods, as well as approaches used for defect detection and classification. This methodology corresponds to the principles of a qualitative critical narrative review, in which the objective is not only to collect the available literature but also to organize, compare, and critically evaluate existing approaches while identifying the main research gaps [37]. Following document-type screening, 307 records (299 research articles and 8 review papers) were retained. After title and abstract screening, 124 records remained. Full-text access was obtained for 103 publications, of which 59 studies were ultimately included in the qualitative synthesis.

## 3. Conveyor Belt Damage Mechanisms

Conveyor belt damage represents a complex engineering problem resulting from the interaction of multiple mechanical, operational, and environmental factors. Unlike many other components of transport systems, conveyor belts operate under continuous contact with transported material, leading to cumulative loading and the gradual degradation of their functional properties [13,15,38,39]. A characteristic feature of conveyor belt degradation is its multi-stage evolution, ranging from local and often difficult-to-detect defect initiation to catastrophic failures resulting in system shutdown.

From a diagnostic perspective, distinguishing between different damage mechanisms is particularly important because they directly determine both the form of degradation and its subsequent representation in diagnostic data. In this study, a physics-based approach to conveyor belt degradation is adopted, enabling the relationship between observed damage forms, diagnostic signals, and appropriate detection methods to be systematically analyzed.

The primary degradation mechanisms of conveyor belts include abrasive wear, impact-related catastrophic damage, cuts and longitudinal rips, material fatigue, and internal structural degradation. These mechanisms may occur simultaneously, and their mutual interaction often accelerates the overall damage process [39].

One of the dominant degradation processes is abrasive wear of the belt covers caused by prolonged contact with transported material. The intensity of this phenomenon depends on material properties such as particle size distribution, hardness, and grain shape, as well as operational parameters including belt speed and transfer point geometry [40,41]. Abrasive wear gradually reduces cover thickness and modifies the surface structure, thereby increasing the belt susceptibility to subsequent forms of damage.

Another important category includes catastrophic damage caused by material impact in loading zones. Depending on the impact energy and material characteristics, such events may lead to local deformation, perforation, or crack initiation within the belt structure [42,43,44,45]. Particularly hazardous conditions occur when the impact load is concentrated over a small area, resulting in local exceedance of the material strength limit.

**Figure 2:**
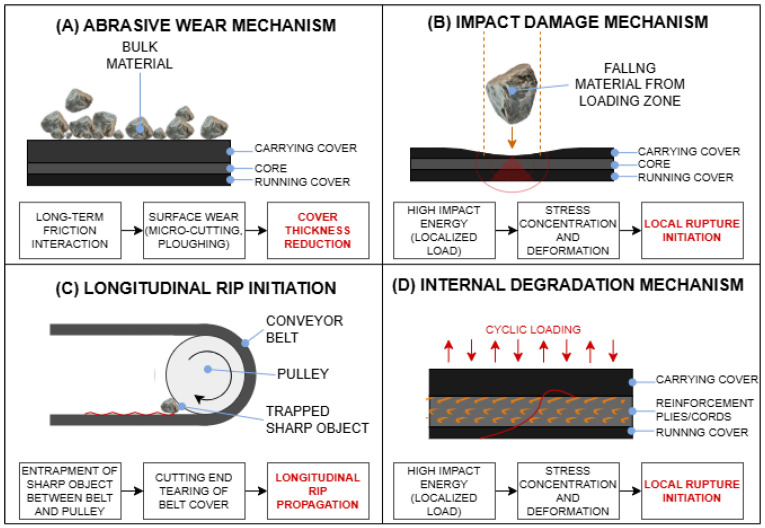
Representative physical mechanisms leading to conveyor belt damage initiation: (**A**) abrasive wear caused by long-term friction interaction with bulk material, (**B**) impact-induced local rupture initiation due to high-energy loading, (**C**) longitudinal rip initiation caused by sharp object entrapment, and (**D**) internal material degradation associated with cyclic loading and fatigue accumulation.

Representative physical mechanisms responsible for conveyor belt damage initiation are schematically illustrated in Figure 2, which presents selected examples of material–belt interactions leading to different forms of degradation.

Surface defects primarily include abrasive wear, cuts, and local deformations [18,46,47,48,49,50]. In many cases, these defects develop gradually and can be monitored using vision-based systems or belt thickness measurements. However, under certain operational conditions, they may evolve into more severe forms of damage, such as longitudinal rips.

Longitudinal rips represent one of the most critical types of conveyor belt damage because they may propagate dynamically and lead to the immediate shutdown of the transport system. Their initiation is frequently associated with sharp-edged material particles or the entrapment of foreign objects within transfer zones [39,43]. Once initiated, the rip may propagate along the belt axis under tensile loading, making early detection particularly important for maintaining system safety and operational reliability.

In addition to material-related degradation mechanisms, conveyor belts are also exposed to operational faults that may initiate or accelerate physical damage. Two particularly important examples are belt looseness or slippage and belt mistracking. Belt looseness may result from insufficient tension, pulley–belt interaction problems, or changes in loading conditions, leading to unstable motion, increased slip, accelerated wear, and reduced transport efficiency. Belt mistracking refers to the lateral deviation in the belt from its intended path. If not detected early, it may cause edge wear, contact with conveyor structures, local overheating, material spillage, and progressive belt damage or failure. Therefore, although looseness and mistracking are not always classified as direct material defects, they represent important operational failure modes that can lead to severe conveyor belt degradation and should be considered in diagnostic reviews.

Belt looseness should be considered as a distinct operational failure mode because it is directly associated with insufficient or unstable belt tension and improper belt–pulley interaction. Unlike localized structural damage, looseness usually does not appear as a single visible defect on the belt surface. Instead, it modifies the dynamic behavior of the conveyor system and may generate secondary symptoms such as slip, intermittent belt–drive engagement, excessive vibration, unstable motion, local impact, increased cover wear, material spillage, and reduced transport efficiency.

The importance of this failure mode is supported by studies addressing belt loading, impact damage, speed variation, dynamic tension behavior, and signal-based fault diagnosis. Andrejiova et al. [42] analyzed impact wear damage of industrial conveyor belts and showed that operating and material-related factors, including drop height, transported material properties, and storage conditions, influence belt wear. Although this study focused on impact wear rather than looseness directly, it demonstrates that unfavorable operating conditions can accelerate belt-cover degradation. Li et al. [51] proposed an online monitoring and fault diagnosis system for belt conveyors based on wavelet packet decomposition and support vector machines, illustrating the usefulness of signal-based feature extraction and machine-learning classification for conveyor fault diagnosis. Zeng et al. [52] investigated the dynamic behavior of a conveyor belt under non-uniform bulk material distribution and showed that variations in transported material distribution affect belt dynamics during speed control. Similarly, He et al. [53] emphasized that inappropriate speed-control transients may introduce operational risks and that healthy conveyor operation requires consideration of dynamic belt behavior during acceleration and deceleration. More recently, Tupkar et al. [54] developed a modelling approach for belt tension and stretch dynamics in medium-duty conveyor systems, further confirming that tension variation and stretch behavior are important parameters for conveyor reliability assessment.

From a diagnostic perspective, the most direct recent contribution is the work of Gelman et al. [55], who proposed non-invasive online technologies for diagnosing conveyor belt looseness using motor current signature analysis. In this approach, looseness is reflected indirectly in the motor current signal through changes caused by intermittent belt–motor engagement and dynamic loading effects. This confirms that belt looseness may be difficult to diagnose using surface-oriented inspection methods alone, while current-signal-based monitoring can provide useful complementary information about belt–drive interaction. However, because current-based diagnostics are indirect, their interpretation requires careful separation of looseness-related effects from load variation, drive dynamics, gearbox condition, speed changes, and other operating disturbances. Consequently, belt looseness should be treated as an important operational failure mode requiring both mechanical understanding of belt tension dynamics and appropriate signal-based diagnostic methods. In addition to surface-visible defects, an important category of degradation mechanisms includes damage developing within the internal belt structure. In multi-layer conveyor belts, these defects primarily involve delamination, reinforcement layer damage, and splice degradation. In steel-cord belts, additional defects include steel cord breakage, deformation, and corrosion [56,57,58,59].

Such defects are particularly difficult to detect because they are often not directly visible on the belt surface. Their development is mainly associated with cyclic loading, local overloads, and environmental influences [13,14,58]. Over extended operating periods, they may lead to a reduction in belt load-carrying capacity and an increased risk of catastrophic failure [31,58].

**Figure 3:**
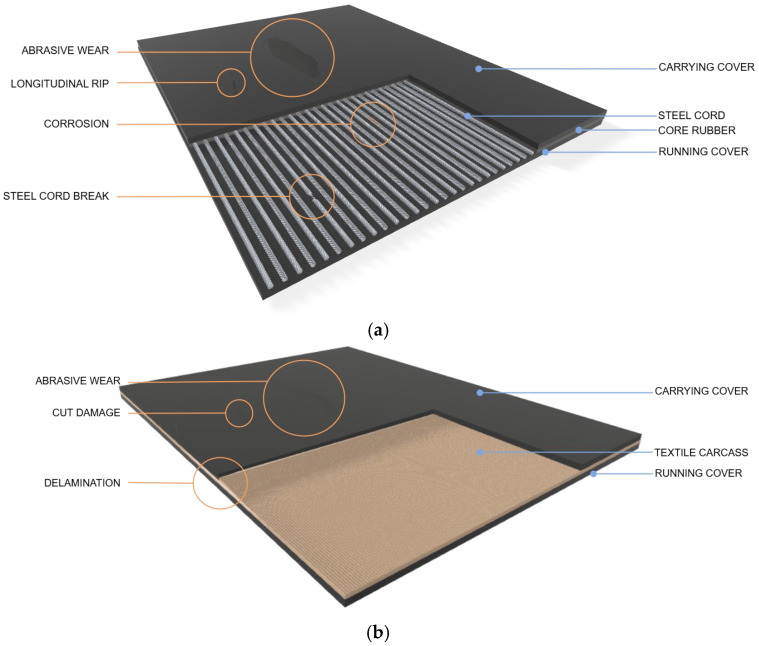
Representative structures and degradation mechanisms of conveyor belts: (**a**) steel-cord belt and (**b**) textile belt. The figure illustrates characteristic construction layers together with selected examples of surface and internal damage mechanisms, including abrasive wear, cut damage, delamination, longitudinal rip, steel cord corrosion, and reinforcement failure.

Figure 3 presents a schematic cross-section of conveyor belts together with the main structural layers and representative damage locations. This visualization helps explain why different diagnostic techniques exhibit sensitivity to different categories of defects.

One of the most important aspects of conveyor belt damage analysis is its representation in diagnostic data. Different degradation mechanisms generate distinct symptoms that can be captured by various sensing and monitoring systems.

Abrasive wear is typically manifested as a gradual reduction in belt thickness and modifications of the surface structure. Catastrophic damage results in local deformations and structural discontinuities, whereas longitudinal rips generate pronounced geometric anomalies. In contrast, steel cord defects are primarily observable in magnetic signals or X-ray images [14].

The analysis of damage mechanisms is therefore fundamental for the design of diagnostic systems. It enables not only the selection of appropriate sensing technologies but also the proper interpretation of acquired data and the identification of relevant diagnostic features. In particular, understanding the relationship between degradation mechanisms and their signal representation creates the possibility of developing more universal analysis approaches that are less dependent on a specific sensing technology. This issue is particularly important in the context of further discussions regarding signal processing and feature extraction methods presented in the following sections of this paper.

## 4. Sensing Technologies for Belt Diagnostics

The selection of sensing technology used in conveyor belt diagnostics depends primarily on the expected damage types, operational conditions, and the nature of diagnostic information required for belt condition assessment. In practice, monitoring systems can be divided into solutions focused mainly on surface defect detection, techniques enabling the evaluation of internal belt structure, and methods indirectly capturing changes in geometry, thickness, or material properties [14,15]. Contemporary conveyor belt diagnostics primarily employ vision-based systems, magnetic methods, ultrasonic techniques, and X-ray imaging, while complementary approaches include thermography, acoustic methods, and systems integrating data from multiple sensing technologies [12,14,15,60].

The largest group of solutions currently used for conveyor belt condition monitoring consists of vision-based systems. Their popularity results mainly from the possibility of non-contact surface inspection during normal conveyor operation and their relatively straightforward integration with existing industrial infrastructure [16,17,18]. These systems are primarily applied for the detection of abrasive wear, cuts, longitudinal rips, and local geometric anomalies [16,17,18,46,47,48,49].

Basic vision systems typically employ line-scan or RGB cameras combined with classical image-processing techniques, including thresholding, segmentation, edge detection, and geometric feature extraction [16,46,61,62]. In recent years, more advanced approaches based on laser scanning, stereoscopic imaging, three-dimensional reconstruction, and spatial data analysis have been rapidly developed [17,48,49,62]. At the same time, a transition from conventional computer vision methods toward deep-learning-based approaches has been observed, enabling the automatic extraction of diagnostic feature representations without the need for manual feature engineering [18,29,32,33,34,35,63].

Despite their high effectiveness in detecting surface defects, vision-based systems exhibit significant limitations in the assessment of damage developing within the internal belt structure and remain highly dependent on environmental conditions [16,17,18]. Diagnostic performance may be significantly affected by dust, surface contamination, varying illumination conditions, and structural vibrations, all of which remain important challenges in industrial implementations [16,17,18].

The diagnostic information provided by vision systems is encoded in image texture, geometric discontinuities, edge structures, and local surface morphology. Abrasive wear is typically associated with gradual texture changes, whereas cuts and longitudinal rips generate distinct geometric features that can be detected using segmentation, contour analysis, or deep-learning-based object detection methods.

Another key group of diagnostic methods includes magnetic techniques based on the Magnetic Flux Leakage (MFL) phenomenon. These methods are particularly important for steel-cord conveyor belt diagnostics because they enable the detection of internal cord defects that are not visible during conventional optical inspection [19,20,21,56,57,58,59].

The operating principle of MFL systems is based on the magnetization of steel cords and the registration of local magnetic field disturbances generated by material discontinuities such as cord breaks, local cross-sectional loss, or progressive degradation processes [19,20,64]. Modern solutions employ multi-channel sensor arrays enabling spatial localization of defects and analysis of diagnostic signal variations as a function of belt position [21,57,59].

The main advantages of magnetic methods include high sensitivity to internal reinforcement defects and the possibility of performing measurements during normal conveyor operation [56,57,58,59]. However, signal interpretation remains strongly dependent on magnetization conditions, measurement system geometry, and the distance between the sensor and belt surface (lift-off effect), which may affect measurement stability [19,20]. An additional limitation is that MFL methods are mainly applicable to steel-cord belts and do not provide direct information about surface damage.

From the data-processing perspective, MFL measurements are typically represented as one-dimensional or multichannel magnetic signals in which defects appear as localized disturbances of the magnetic field. Cord breaks usually generate high-amplitude peaks, whereas corrosion-related degradation often manifests as broader signal distortions with lower amplitudes. Consequently, signal preprocessing commonly includes filtering, baseline correction, lift-off compensation, and feature extraction based on amplitude, energy, spatial extent, or spectral characteristics.

Another important category of diagnostic approaches includes ultrasonic techniques, which are primarily used for evaluating belt thickness, material degradation, and cover wear [22,23,24]. Unlike magnetic methods, ultrasonic systems mainly focus on detecting changes in material properties and local geometric variations associated with degradation processes [22,65,66].

Industrial ultrasonic systems usually employ arrays of transducers positioned on both sides of the moving belt, enabling continuous local thickness measurements and determination of the belt cross-sectional profile [24]. The obtained information is used for wear-rate assessment, maintenance planning, and operational decision support [23,24]. The main limitations of ultrasonic techniques include the need to maintain appropriate measurement conditions and the strong dependence of measurement quality on diagnostic system geometry [22,65].

Ultrasonic measurements are commonly represented as thickness profiles or echo signals. Diagnostic information is contained in signal amplitude, time-of-flight, attenuation characteristics, and local discontinuities. These parameters can be further processed using statistical, spectral, or time–frequency analysis methods to quantify material degradation and wear progression.

A further important class of conveyor belt monitoring methods is based on motor current signature analysis (MCSA) and current-signal-based diagnostics. Unlike vision, MFL, ultrasonic, or X-ray systems, these methods do not observe the belt surface or internal structure directly. Instead, they use current signals acquired from the drive motor or gearmotor to identify load-related anomalies associated with abnormal conveyor operation. This approach is attractive for industrial applications because current data can often be acquired from the electrical drive system without installing additional sensors along the conveyor route. Farhat et al. [67] proposed an online diagnostic method for conveyor belt mistracking based on MCSA. The method used higher-order spectral features, including bicoherence, tricoherence, and the cross-correlation of spectral moduli of order 3, to detect changes in motor current caused by increased friction between the mis-tracked belt and conveyor structure. Gelman et al. [68] also demonstrated conveyor belt monitoring using current signals, showing that features related to the fundamental harmonic of the motor supply frequency can be used for belt tracking diagnosis. In addition to mistracking detection, MCSA has recently been applied specifically to conveyor belt looseness diagnosis. Gelman et al. [55] proposed online non-invasive diagnostic technologies in which looseness-related changes are detected from motor current signals. This direction is important because belt looseness modifies the belt–drive interaction and may generate current-signal symptoms before the failure becomes directly visible in surface inspection data. Therefore, MCSA can complement vision, MFL, ultrasonic, and X-ray methods by capturing operational failure modes related to belt tension, slip, and dynamic loading. These studies indicate that MCSA-based monitoring may complement direct belt inspection methods, particularly for detecting operational faults such as mistracking, slippage, or abnormal load conditions. However, such methods usually provide indirect diagnostic information and require careful interpretation, because motor current variations may also be influenced by load changes, drive dynamics, gearbox condition, and operating regime.

A separate class of solutions is represented by X-ray imaging systems, which are primarily used for the inspection of the internal structure of steel-cord conveyor belts [25,26,27]. X-ray methods enable direct observation of steel cord arrangement, identification of reinforcement defects, and assessment of selected structural discontinuities that remain undetectable using surface-based inspection techniques [25,26].

The increasing interest in automated interpretation of X-ray images has recently led to the growing application of deep-learning-based approaches supporting automatic defect identification [27,69]. These methods increase the potential for fully automated diagnostics; however, their implementation remains limited by equipment cost, radiological safety requirements, and the higher complexity of industrial installations [25,26,27].

Unlike MFL and ultrasonic measurements, X-ray systems generate image-based representations of the internal belt structure. Defects are typically expressed as local structural anomalies, missing reinforcement elements, or splice irregularities. As a result, image enhancement, segmentation, object detection, and deep-learning-based feature extraction constitute essential stages of the diagnostic workflow.

**Figure 4:**
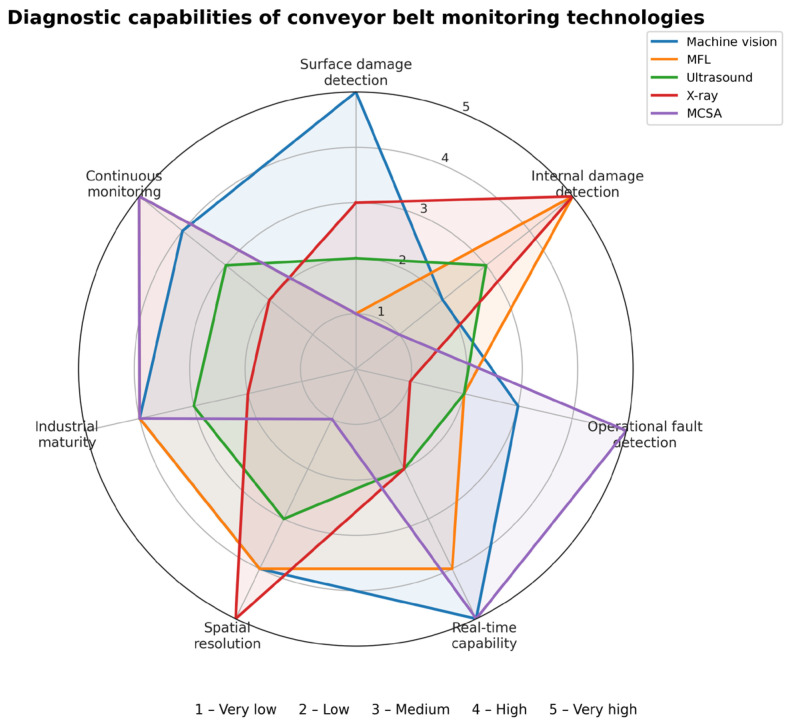
Qualitative comparison of diagnostic capabilities of selected sensing technologies applied in conveyor belt diagnostics. Radar chart illustrating relative strengths of machine vision systems, magnetic flux leakage (MFL), ultrasonic techniques, X-ray imaging, and motor current signature imaging considering surface damage detection, internal damage detection, continuous monitoring capability, industrial maturity, spatial resolution, and real-time applicability.

To provide a synthetic comparison of the diagnostic capabilities of different sensing technologies, a qualitative assessment was developed considering surface and internal defect detection capability, suitability for continuous operation, industrial maturity, spatial resolution, and real-time diagnostic potential. As illustrated in Figure 4, the analyzed technologies exhibit complementary diagnostic properties, highlighting the potential benefits of integrating multiple sensing modalities.

**Figure 5:**
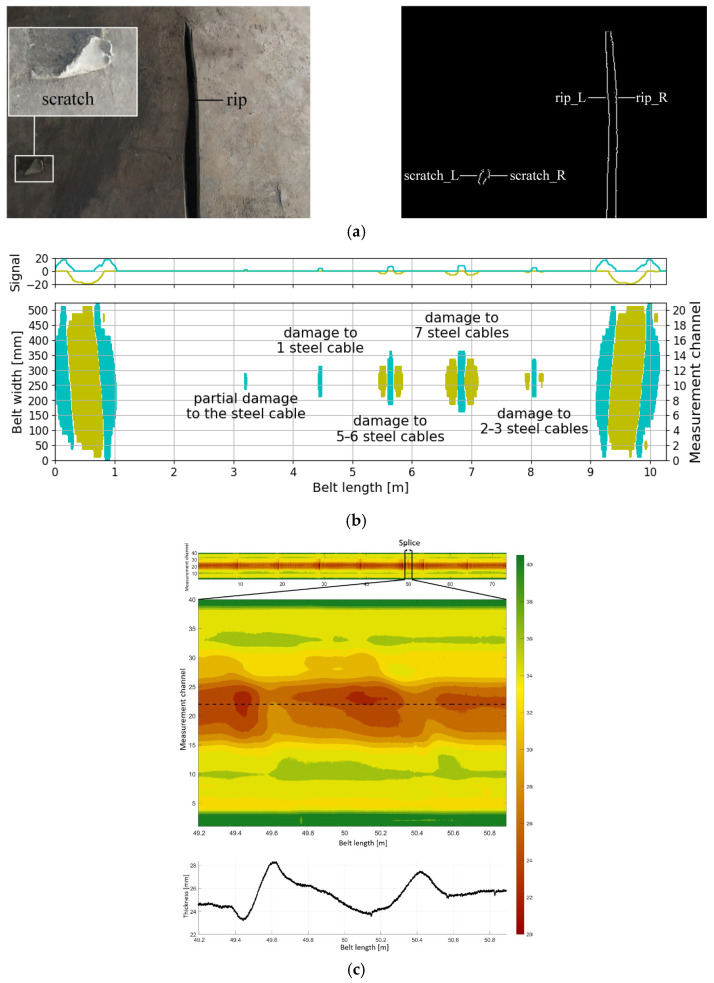
Representative examples of diagnostic data generated by different sensing technologies used in conveyor belt monitoring: (**a**) vision-based surface image showing a longitudinal rip [48], (**b**) MFL signal with a localized magnetic anomaly associated with steel cord damage, (**c**) ultrasonic thickness profile indicating cover wear.

While Figure 4 summarizes the general diagnostic capabilities of selected sensing technologies, their practical usefulness strongly depends on the type of data generated and the way damage-related information is encoded in these data. From the perspective of signal-based diagnostics, the key issue is therefore not only whether a given technology can detect a defect, but also how this defect is represented in the acquired signal or image. Representative examples of diagnostic data generated by different sensing technologies are shown in Figure 5.

The examples presented in Figure 5 illustrate that different sensing technologies generate fundamentally different forms of diagnostic data. Consequently, the effectiveness of damage detection depends not only on the sensing modality itself but also on the way diagnostic information is represented and subsequently processed. To facilitate the comparison of these approaches from a signal-processing perspective, Table 1 summarizes the characteristic data representations, defect signatures, and processing methods associated with the most commonly applied sensing technologies.

As shown in Table 1, conveyor belt diagnostic systems differ considerably in the type of information they provide and in the signal-processing procedures required for damage identification. While machine vision and X-ray systems rely primarily on image analysis techniques, MFL and ultrasonic methods generate one-dimensional signals or profiles that are typically analyzed using statistical, spectral, or time–frequency approaches. These differences motivate the need for a more detailed discussion of signal-processing methods presented in the following section.

Although the diagnostic capabilities of individual sensing technologies are commonly compared in terms of defect detectability, their industrial applicability is also limited by specific failure modes and sensitivity degradation mechanisms. These limitations are particularly important in harsh operating environments, where dust, surface contamination, vibration, belt speed variation, unstable sensor geometry, and limited maintenance access may significantly affect measurement repeatability. Table 2 summarizes the main industrial failure modes of conveyor belt sensing technologies and their expected influence on diagnostic sensitivity.

The comparison shows that the practical performance of conveyor belt diagnostic systems cannot be evaluated only by nominal detection capability. For example, machine-vision systems may achieve high accuracy under controlled conditions, but their sensitivity to dust, illumination variability, and surface contamination remains a major source of false detections and missed defects. Similarly, MFL systems are well suited to steel-cord belt diagnostics, but their reliability depends strongly on maintaining stable lift-off, magnetization, and sensor-head orientation. Consequently, future studies should report not only classification accuracy or mAP, but also robustness indicators under controlled environmental disturbances, such as illumination variation, dust contamination, sensor-to-belt distance changes, vibration, belt speed variation, and long-term calibration drift.

The conducted analysis indicates that none of the currently applied sensing technologies can be considered a universal solution. Vision-based systems dominate in the detection of surface defects, magnetic methods remain the primary diagnostic tool for assessing the condition of steel-cord belt reinforcement, ultrasonic techniques are mainly used for monitoring material degradation and wear, whereas X-ray imaging provides the most direct information regarding the internal belt structure [14,15].

Consequently, the current development of conveyor belt diagnostics is increasingly focused not on replacing one sensing technology with another, but rather on integrating multiple data modalities and developing more comprehensive monitoring systems based on multisensor fusion strategies and advanced data-analysis methods [60,70,71,72].

## 5. Signal Representation and Processing

The effectiveness of diagnostic systems used for conveyor belt condition monitoring depends not only on the applied sensing technology but also on the representation and processing of the acquired data. Individual diagnostic techniques do not detect damage directly; instead, they register its physical consequences, such as surface texture modifications, local geometric deformations, magnetic field disturbances, thickness variations, or anomalies within the internal structure of the belt [14,15,19]. Consequently, the same degradation mechanism may generate different diagnostic representations depending on the sensing technology used.

For example, a longitudinal rip may be represented as a geometric anomaly in vision-based systems [46,47,48,49], a local thickness variation in ultrasonic measurements [22,23,24] or a structural discontinuity in X-ray imaging [25,26,27]. Similarly, steel cord defects generate characteristic disturbances in Magnetic Flux Leakage (MFL) signals [19,20,21,56,57,58,59], while often remaining invisible during conventional optical inspection.

The diagnostic process usually consists of several fundamental stages, including data acquisition, preprocessing, feature extraction, dimensionality reduction, and diagnostic classification or interpretation [9,10,11,28,29]. In recent years, a transition from classical handcrafted feature extraction toward approaches based on automatic representation learning using deep learning methods has also been observed [29,31,32,33,34,35,69].

One of the major challenges in conveyor belt diagnostics remains the lack of a universal damage representation enabling direct comparison of results obtained using different sensing technologies. Individual diagnostic systems register different physical manifestations of the same degradation process, meaning that an identical defect may assume significantly different diagnostic representations.

In vision-based systems, defect representation is primarily based on the analysis of texture, geometry, and optical surface properties [16,17,18,46,47,48,49,50]. Abrasive wear typically results in gradual texture modification, whereas cuts and longitudinal rips generate local geometric discontinuities detectable using image segmentation, contour analysis, or three-dimensional data processing methods [48,49].

Magnetic methods based on Magnetic Flux Leakage (MFL) represent damage through local magnetic field disturbances generated by discontinuities in steel cords [19,20,21,56,57,58]. Signal characteristics depend on factors including defect type, defect size, spatial location, and magnetization conditions [19,21,73].

Ultrasonic techniques primarily utilize local variations in material thickness, reflected signal amplitude, and wave propagation time [22,23,24]. In contrast, X-ray imaging enables direct representation of the internal belt structure and is particularly important in the diagnostics of steel-cord conveyor belts [25,26,27].

Table 3 presents examples of conveyor belt defect representation in different sensing modalities.

While Table 3 highlights how the same defect may be represented differently depending on the sensing modality, the selection of signal-processing methods is equally dependent on the type of diagnostic information available. Different damage mechanisms generate distinct signal characteristics, requiring dedicated preprocessing, feature extraction, and analysis strategies. To provide a more application-oriented perspective, Table 4 summarizes the relationships between common conveyor belt defects, their diagnostic representations, and the signal-processing methods most frequently employed for their identification.

As shown in Table 4, the suitability of individual signal-processing methods depends strongly on both the sensing technology and the physical manifestation of the defect. Localized discontinuities, such as steel cord breaks or longitudinal rips, often require time–frequency or geometric analysis techniques capable of capturing local signal variations, whereas gradual degradation processes, including abrasive wear, can frequently be characterized using statistical, texture-based, or trend-analysis features. Consequently, the choice of processing methodology should be guided by the expected damage morphology and data representation rather than by the sensing technology alone.

Data preprocessing represents one of the key stages of the diagnostic process because the quality of input data directly affects the effectiveness of subsequent feature extraction and classification stages [29,74,75,76,77].

In vision-based systems, preprocessing mainly includes noise filtering, brightness normalization, contrast enhancement, thresholding, and image segmentation [16,17,18,46,47,48,49,50]. Reducing the influence of variable illumination conditions, dust, and surface contamination is particularly important, as these factors constitute significant challenges for industrial diagnostic systems operating in harsh environments [16,17].

For one-dimensional signals such as MFL signals, thickness profiles, or ultrasonic measurements, preprocessing procedures typically involve filtering and noise reduction techniques including signal smoothing, median filtering, and normalization [19,21,30].

Particularly important are time–frequency analysis methods used for the investigation of non-stationary signals. The most commonly applied approaches include the Fourier Transform (FFT), Short-Time Fourier Transform (STFT), wavelet transform, and Empirical Mode Decomposition (EMD) [29,74,75,76]. These methods enable the identification of local signal characteristic variations associated with developing damage.

The suitability of individual signal-processing methods depends strongly on both the sensing technology and the damage type under investigation. FFT-based methods are mainly applied when periodic or frequency-related characteristics are of interest, whereas wavelet transforms and EMD are particularly useful for non-stationary signals containing localized disturbances, such as those generated by steel-cord defects in MFL measurements. Similarly, STFT provides a compromise between time and frequency resolution and is often used for the analysis of transient diagnostic events. Consequently, no single processing method can be considered universally optimal for conveyor belt diagnostics, and method selection should be guided by the physical characteristics of both the measured signal and the expected defect.

### 5.1. Diagnostic Feature Extraction

Diagnostic feature extraction is the process of transforming raw data into representations enabling effective defect identification [28,29].

The most commonly used group consists of statistical features including mean value, standard deviation, RMS, variance, skewness, and kurtosis [9,28]. Such parameters are particularly widely applied in condition monitoring and one-dimensional signal diagnostics [9,10,11].

In vision-based systems, geometric features are extensively used and include rip length, cut width, damaged area, and parameters describing object geometry [46,47,48].

Increasing attention is also being devoted to texture features used for describing local surface structure. Particularly common approaches include the Grey-Level Co-occurrence Matrix (GLCM) [78], Local Binary Patterns (LBP) [79] and entropy-based measures [18,50].

Signal analysis additionally employs frequency-domain and time–frequency features including band energy, dominant frequencies, and wavelet coefficients [29,75,76].

Feature effectiveness also depends on the underlying degradation mechanism. Statistical descriptors are often sufficient for monitoring gradual wear processes, whereas localized defects such as cuts, rips, or cord breaks usually require geometric, texture-based, or time–frequency features capable of capturing local signal variations. As a result, feature-selection strategies should be adapted to the expected damage morphology rather than applied uniformly across all diagnostic tasks.

### 5.2. Learned Feature Representations

Classical diagnostic approaches are mainly based on handcrafted features, whose effectiveness strongly depends on expert knowledge and the appropriate selection of analysis parameters [28].

In recent years, however, the rapid development of deep-learning-based approaches enabling automatic feature representation learning directly from raw input data has been observed [29,69].

Convolutional Neural Networks (CNNs) are particularly widely applied because they enable automatic detection of local spatial patterns and the construction of hierarchical data representations [69,80]. In conveyor belt diagnostics, CNN-based solutions are used for surface defect classification, longitudinal rip detection, and wear identification [18,32,33,34,35].

Growing interest is also being observed in transfer learning methods, autoencoders, and approaches enabling multimodal data integration [71,72].

### 5.3. Challenges Related to Data Representation

Despite significant advances in diagnostic methods, a unified approach to damage representation across different sensing technologies is still lacking. Most currently developed methods are designed for a single data modality, which limits both the comparability of results and the transferability of developed solutions [71,72].

Another important limitation is the restricted availability of standardized datasets enabling the objective evaluation of proposed methods and supporting the development of more universal classification algorithms [11,29].

Consequently, one of the most promising future research directions involves the development of a shared feature representation space enabling the integration of data originating from different sensing technologies and the construction of more universal diagnostic systems.

**Figure 6:**
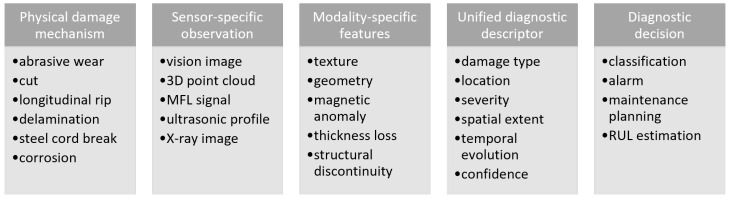
Conceptual framework for unified conveyor belt damage representation. Sensor-specific observations generated by different sensing technologies are first transformed into modality-specific features and then mapped onto a common diagnostic descriptor space, enabling comparison, fusion, and maintenance-oriented decision-making.

To make the concept of unified damage representation more actionable, it is useful to distinguish between sensor-specific data representation and diagnostic-level damage representation. Different sensing technologies generate different raw data structures, such as RGB images, 3D point clouds, MFL signals, ultrasonic thickness profiles, or X-ray images. However, after preprocessing and feature extraction, these data can be mapped onto a common diagnostic descriptor space. Such a space may include damage class, location along and across the belt, severity, spatial extent, temporal evolution, data quality, and diagnostic uncertainty. In this interpretation, unified damage representation does not require different sensing systems to produce identical raw data. Instead, it requires that their outputs be transformed into comparable diagnostic descriptors that can support benchmarking, multimodal fusion, and maintenance-oriented decision-making. A conceptual framework illustrating this transformation from physical damage mechanisms to diagnostic decisions is presented in Figure 6.

Such a framework may serve as a basis for future conveyor belt diagnostic systems in which different sensing modalities remain technically independent at the acquisition level but become comparable at the diagnostic interpretation level.

## 6. Damage Detection and Classification Methods

The effectiveness of conveyor belt monitoring systems depends not only on the applied sensing technology but also on the methods used for diagnostic data interpretation. Regardless of the sensor type, the diagnostic process typically involves anomaly detection, feature extraction, and assignment of observed changes to specific damage classes [9,10,11,29]. Along with the increasing availability of diagnostic data and the rapid development of artificial intelligence methods, a gradual transition from rule-based approaches toward automatic representation learning techniques has been observed [29,69].

The approaches used in conveyor belt diagnostics can generally be divided into five main categories:

- Conventional signal and image processing methods,
- Supervised classical machine learning algorithms,
- Unsupervised and one-class anomaly detection methods, deep learning approaches,
- Hybrid and multimodal systems.

### 6.1. Conventional Signal and Image Processing Approaches

The earliest solutions used in conveyor belt diagnostics were mainly based on classical image and signal processing methods. Their primary advantages include relatively low computational requirements and good interpretability of results [16,17].

In vision-based systems, commonly applied techniques include thresholding, edge detection, morphological operations, and contour analysis [16,46,61]. Particularly popular are longitudinal rip detection algorithms based on the analysis of damage geometry and local surface texture variations [46,47,48,49].

A representative example of a geometry-oriented approach was presented by Xu et al. [48], who proposed longitudinal rip detection based on 3D point cloud processing. Instead of relying only on pixel intensity or surface texture, the method used a binocular line-laser stereo vision system to acquire spatial point data from the belt surface. Suspected defect points were extracted from the point cloud, clustered, and then discriminated to distinguish true longitudinal rips from scratches. Principal component analysis was subsequently used to characterize the direction and maximum width of the rip. The method achieved a correct identification rate of 99.2%, with an identification time below 0.04 ms and a relative error of rip-width estimation within ±5%. This study illustrates that geometric 3D representations can reduce false alarms caused by surface scratches and illumination variability.

Edge detection methods are widely used for identifying cuts, longitudinal rips, and local surface deformations. In industrial applications, Sobel, Prewitt, and Canny operators are among the most commonly applied approaches [61].

For one-dimensional signal analysis, threshold-based methods, amplitude analysis, spectral analysis, and time–frequency techniques are widely employed [28,74,75,76]. In diagnostics based on Magnetic Flux Leakage (MFL) signals, particular importance is attributed to the analysis of local amplitude variations and frequency characteristics associated with steel cord defects [19,20,21].

Despite their relatively high interpretability, conventional approaches exhibit limited robustness to environmental variability and remain highly sensitive to the selection of analysis parameters [16,18].

### 6.2. Machine Learning-Based Damage Classification

The development of condition monitoring systems has led to a gradual increase in the use of classical machine learning methods in industrial diagnostics [9,10,11].

In such approaches, the diagnostic process usually consists of data preprocessing, handcrafted feature extraction, and application of a classification algorithm [10,11]. A summary of representative classical machine-learning methods relevant to conveyor belt diagnostics is presented in Table 5.

The suitability of individual machine-learning methods depends strongly on dataset size, feature quality, and diagnostic objectives. SVM-based classifiers are particularly attractive in conveyor belt diagnostics because they often provide competitive performance when only limited datasets are available. In contrast, ensemble methods such as Random Forest offer improved robustness to noisy measurements but may provide less insight into the physical relevance of individual diagnostic features. Consequently, classical machine-learning approaches remain particularly valuable in industrial environments where data availability is limited and model interpretability is important.

The most commonly used methods include Support Vector Machines (SVM), Random Forest (RF), k-Nearest Neighbours (k-NN), and classical Artificial Neural Networks (ANN) [10,29].

These approaches enable effective damage classification even for relatively limited datasets; however, their performance strongly depends on the quality of the feature extraction process [10]. Consequently, the development of representative feature spaces remains one of the key challenges of classical diagnostic systems.

In addition to supervised classification methods, an important group of diagnostic approaches is based on unsupervised or one-class anomaly detection. These methods are particularly relevant for industrial conveyor belt monitoring because labelled examples of belt faults are often scarce, whereas data representing normal or healthy operating conditions can usually be collected during routine operation [9,10,11,29,81]. In this approach, the diagnostic model learns a representation of the normal belt or conveyor-system state, and observations that deviate significantly from this learned normal state are treated as anomalies.

One-class methods are useful when fault data are unavailable or highly imbalanced. One-Class Support Vector Machine models can be trained using only healthy-condition feature vectors and then used to identify observations located outside the learned normal-data boundary [82]. In conveyor belt diagnostics, such feature vectors may be extracted from MFL signals, ultrasonic thickness profiles, motor current signals, vibration data, or image-based descriptors. This type of approach is attractive because it does not require explicit examples of every possible damage mechanism during training.

Autoencoder-based anomaly detection provides another important direction. Autoencoders learn to reconstruct normal input data, and the reconstruction error can be used as an anomaly score [83,84]. If the model is trained primarily or exclusively on healthy-condition data, damaged or abnormal states are expected to produce higher reconstruction errors because they differ from the learned normal representation. Depending on the sensing modality, autoencoders may be applied to one-dimensional diagnostic signals, multichannel MFL data, current-signal features, ultrasonic profiles, or image patches acquired from the belt surface. Convolutional autoencoders are particularly suitable for image-based or spatial data, whereas fully connected or sequence-based autoencoders may be used for signal and profile data.

For time-dependent diagnostic data, recurrent models such as LSTM networks and LSTM autoencoders are especially relevant. They can learn normal temporal patterns of conveyor operation and detect deviations associated with abnormal load conditions, belt mis-tracking, slippage, sensor drift, or developing damage [85]. Such approaches are well suited to continuous monitoring systems because they can model temporal dependencies in operational data, including motor current, belt speed, vibration, or periodically acquired sensor signals.

The main advantage of unsupervised and one-class anomaly detection is that it reflects a realistic industrial situation in which healthy-condition data are abundant, while representative fault datasets are incomplete or unavailable. However, this approach also has limitations. First, anomaly detection usually indicates that the current state deviates from normal operation, but it does not always directly identify the damage type or physical cause. Second, models trained on healthy data may generate false alarms when operating conditions change, for example, due to variable loading, belt-speed variation, environmental disturbances, sensor drift, or maintenance interventions. Therefore, robust threshold selection, operating-regime normalization, and periodic model updating are essential. In practical conveyor belt diagnostics, unsupervised anomaly detection should be treated as complementary to supervised classification and object detection: it is particularly useful for early warning and novelty detection, while supervised or rule-based methods remain important for fault attribution and maintenance decision-making.

### 6.3. Deep Learning Approaches

In recent years, rapid development of deep learning methods applied to industrial diagnostics has been observed [29,69]. Unlike classical machine learning approaches, deep learning models enable automatic extraction of feature representations directly from raw input data.

Convolutional Neural Networks (CNNs) are particularly widely used because they allow automatic analysis of local spatial structures present in image data [69,80].

Instead of treating deep-learning methods as a homogeneous group, representative studies should be analyzed according to diagnostic task, input data type, model architecture, and reported performance. The following examples illustrate how recent conveyor belt diagnostic studies have moved from generic CNN-based classification toward task-specific architectures designed for longitudinal rip localization, wear-state assessment, X-ray-based internal defect detection, and lightweight real-time monitoring.

An important development direction also involves object detection architectures such as YOLO [86], which enable real-time defect localization. In applications requiring accurate defect segmentation, U-Net-based architectures are increasingly employed [87].

Object-detection architectures are particularly relevant for conveyor belt diagnostics because they provide not only image-level classification but also spatial localization of damage. For example, Wang et al. [86] proposed YOLOX-BTFPN, an anchor-free conveyor belt damage detector with a biassed texture feature pyramid network designed to better capture belt surface damage characteristics. The method used decoupled detection heads and a SimOTA assignment strategy to improve detection reliability and convergence. Reported mAP values reached 98.45% and 94.37% on two test datasets, respectively, demonstrating the suitability of task-specific object detectors for conveyor belt damage localization.

Zhou et al. [32] provided a representative example of a task-specific deep-learning architecture for conveyor belt wear assessment. The authors combined multispectral imaging in the 675–975 nm range with a lightweight network named DSCANet to classify conveyor belts into three wear states: no wear, normal wear, and severe wear. A shallow CNN was first used for wavelength screening, and the multispectral image at 722 nm, which provided the highest average F1-score during wavelength selection, was used as the final model input. The proposed DSCANet was derived from EfficientNetB0 but modified by introducing DSCA-MBConv blocks, group convolution, channel shuffle, GELU activation, and a novel depthwise shuffle coordinate attention module designed to focus the network on spatially irregular wear regions. The model achieved 98.78% accuracy, 98.78% precision, 98.77% recall, and 98.77% F1-score, with only 136.53 M FLOPs, outperforming EfficientNetB0, GhostNet, MobileNetV2, MobileNetV3, ShuffleNetV2, ResNet50, and DenseNet169. This study is important because it shows that wear assessment requires not only image classification but also spectral band selection, attention mechanisms, lightweight architecture design, and robustness evaluation under noise disturbance.

Recent studies demonstrate that deep-learning approaches in conveyor belt diagnostics are increasingly moving from generic CNN classification toward task-specific detection architectures. For example, Wang et al. [27] proposed an improved FCOS-based detector for X-ray images of steel-cord conveyor belts. The method addressed the long and narrow geometry of belt damage by introducing CIoU loss for bounding-box regression and adaptive paths in the feature fusion network. Compared with the original FCOS model, the improved method increased precision and recall by 20.9% and 14.8%, respectively, reaching 95.8% precision and 97.0% recall. This example illustrates that architecture adaptation to the specific geometry of conveyor belt defects may be more important than the direct application of generic object detectors.

Another important research direction concerns training-data limitations. Guo et al. [18] proposed an MCC-CycleGAN framework for conveyor belt surface damage detection, combining two generators, two discriminators, and a ResNet-34-based critic network for multi-class classification. The model used generated samples and an incremental image fusion strategy to reduce overfitting caused by limited training data. The reported mAP and Macro-F1 reached 96.9% and 96.8%, respectively, clearly outperforming conventional CNN backbones such as ResNet-34, ResNet-50, VGG16, Inception v3, and AlexNet. This shows that generative augmentation may be particularly valuable in conveyor belt diagnostics, where industrial defect datasets are usually small and imbalanced.

Lightweight object-detection models are also increasingly important for real-time conveyor belt monitoring. Guo et al. [50] proposed a machine-vision-based damage detection method using CenterNet combined with fusion knowledge distillation. The method introduced feature-wise and response-wise knowledge distillation to balance model accuracy and computational cost, while a fused channel-spatial attention mechanism was used to compress latent feature maps efficiently. The resulting lightweight detector achieved 92.53% mAP and 65.8 FPS, indicating its potential for deployment on end devices and online conveyor belt monitoring systems.

A recent 2025 study further confirms the importance of architecture adaptation and industrial validation. Li et al. [88] proposed a laser-scanning-based micro-damage detection system combined with an improved YOLOv7 model incorporating F-ReLU activation, the SPPFCSPC module, EIoU loss, and the SE-Net attention mechanism. The model achieved mAP@0.5 of 96.6%, compared with 90.7% for the baseline YOLOv7, and was additionally evaluated during 72 h continuous operation and low-light industrial deployment. This study indicates that future conveyor belt diagnostic systems should be evaluated not only by classification accuracy but also by robustness, inference speed, and stability under real industrial conditions.

Although deep-learning models frequently achieve the highest classification accuracies reported in the literature, their practical deployment remains associated with several challenges. CNN-based models generally require large annotated datasets and substantial computational resources, while their performance may deteriorate when operating conditions differ significantly from those represented in the training data. Object-detection architectures such as YOLO offer real-time processing capabilities, making them attractive for online monitoring; however, their effectiveness depends strongly on image quality and the availability of representative training examples. Similarly, segmentation-oriented architectures such as U-Net provide detailed localization of defects but require pixel-level annotations, which are often difficult and expensive to obtain in industrial environments.

Growing interest is additionally observed in transfer learning, self-supervised learning, transformer-based models [89], and reconstruction-based anomaly detection approaches, which may help mitigate the limitations associated with small or incomplete fault datasets [81,83,89].

### 6.4. Hybrid and Multimodal Approaches

Despite the rapid development of deep learning methods, most currently applied diagnostic systems remain strongly dependent on a single sensing technology [71,72].

However, increasing attention is being devoted to hybrid approaches integrating data originating from multiple sensing modalities. Combining information obtained from vision systems, magnetic methods, and geometric measurements improves diagnostic robustness and reduces the risk of incorrect classification [71,72,90].

A particularly important research direction involves multimodal data fusion approaches enabling the integration of heterogeneous data obtained from different sensing modalities [71,72]. Existing studies have demonstrated the feasibility of combining visual information with geometric measurements, MFL signals, or thickness profiles to improve defect detection robustness and reduce false-alarm rates. However, most currently reported solutions remain limited to laboratory-scale demonstrations, and large-scale industrial implementations are still scarce.

Several conveyor-belt-oriented studies have already demonstrated the potential of multimodal or multisensor fusion, although most of them focus on longitudinal tear detection rather than on a fully integrated diagnostic framework. Qiao et al. [91] proposed an integrative binocular vision detection method based on infrared and visible-light image fusion. In this approach, fused images were used for tear-feature extraction and identification, and the reported average detection accuracy reached approximately 96%, with image-processing time below 18 ms. Che et al. [92] developed an audio–visual fusion method in which a visible-light CCD camera and a microphone array were used to collect image and sound data from conveyor belts operating in different states. Image and sound features representing normal, scratch, and longitudinal-tear conditions were then fused and classified using a machine-learning algorithm; the reported longitudinal-tear detection accuracy exceeded 96%. More recently, Wang et al. [93] proposed a multi-mode fusion method combining visual information from a linear CCD camera with acoustic signals acquired by a microphone array. In this approach, visual data were classified using an improved ShuffleNet_V2 network, acoustic features were classified using a CNN-LSTM model, and final decision fusion was performed using Dempster–Shafer theory.

These examples show that multimodal fusion can improve diagnostic robustness by combining complementary spatial, optical, thermal, and acoustic information. However, they also indicate that existing conveyor belt fusion studies are still concentrated mainly on vision-based or audio–visual tear detection. Fully integrated heterogeneous systems combining machine vision, MFL, ultrasonic measurements, and operational data within a single diagnostic architecture remain rare. Therefore, multimodal fusion should be regarded as a partially developed but still open research direction in conveyor belt diagnostics.

### 6.5. Edge AI, Explainable AI, and Digital-Twin-Based Diagnostic Frameworks

The practical deployment of conveyor belt diagnostic systems increasingly requires not only accurate defect classification but also real-time processing, transparent decision-making, and integration with maintenance planning. For this reason, recent research directions extend beyond individual detection algorithms toward Edge AI, Explainable Artificial Intelligence, and Digital-Twin-based diagnostic frameworks.

Edge AI is particularly relevant for conveyor belt monitoring because diagnostic systems are often distributed over long transport routes and generate large streams of image, magnetic, ultrasonic, or operational data. In conventional cloud-oriented architectures, large raw data streams must be transmitted to centralized servers, which may increase latency, communication load, and dependence on network availability. In contrast, edge-based architectures enable part of the preprocessing, feature extraction, and diagnostic inference to be performed close to the measurement system [94]. This is especially important for belt monitoring systems requiring fast alarm generation after local damage initiation. Recent reviews on TinyML and edge intelligence in Industrial Internet of Things applications emphasize that lightweight models deployed on microcontrollers, gateways, or industrial edge computers can support predictive maintenance, equipment monitoring, anomaly detection, and quality inspection while reducing data transmission requirements and improving local responsiveness [95]. In conveyor belt diagnostics, this trend is reflected by the increasing use of lightweight architectures, knowledge-distillation-based detectors, and optimized object-detection models that balance accuracy, inference speed, and computational complexity.

Explainable Artificial Intelligence represents another important requirement for industrial deployment. In conveyor belt diagnostics, maintenance personnel usually require not only information that a defect has been detected but also an explanation of why the system produced a given decision. Recent XAI research in industrial fault detection and diagnosis emphasizes that the lack of transparency of advanced machine-learning and deep-learning models reduces trust in safety-critical applications and limits their practical acceptance [96]. For image-based conveyor belt diagnostics, Grad-CAM and saliency-map methods can help verify whether CNN or object-detection models focus on actual damage regions rather than background artefacts, dust, illumination changes, or irrelevant belt texture. This is particularly relevant for wear assessment models using attention mechanisms, where activation maps can indicate whether the model concentrates on worn regions. For signal-based diagnostics, SHAP- or LIME-type explanations may support the interpretation of feature importance in MFL, ultrasonic, or thickness-profile data [96]. Therefore, XAI methods should be considered not only as visualization tools but also as mechanisms supporting trust, auditability, and operator-oriented maintenance decisions.

Digital Twin technologies provide a broader framework for integrating diagnostic data, degradation models, and maintenance decisions. In conveyor belt diagnostics, a digital twin can be defined as a dynamic virtual representation of the belt system continuously updated using sensing and operational data. Such a framework may integrate information from vision systems, MFL measurements, ultrasonic thickness profiles, belt speed, loading conditions, environmental parameters, and maintenance history. The collected data can then be processed to estimate the current damage state, update degradation models, forecast remaining useful life, and support maintenance planning [97,98,99].

Recent conveyor-belt-oriented studies have already demonstrated the feasibility of this direction. Pulcini and Modoni [97] proposed a machine-learning-based digital twin of a conveyor belt for predictive maintenance in a real-case scenario. In their approach, the core of the digital twin was a machine-learning model analyzing data collected from sensors distributed along the conveyor belt in order to predict fault occurrence during normal operation. Mafia et al. [98] proposed a digital twin design for conveyor belt predictive maintenance in which different sensors collect heterogeneous data to be analyzed by a digital system. Their framework emphasized the role of simulation and integration with the physical system, including the possibility of generating additional data to reduce the time required to train AI algorithms. More generally, recent manufacturing-oriented reviews indicate that AI-driven digital twins are evolving from passive virtual representations toward real-time monitoring, predictive analytics, and autonomous decision-support systems [99].

A conceptual conveyor-belt-oriented digital twin architecture should therefore include several interconnected layers: a sensing layer acquiring image, magnetic, ultrasonic, geometric, and operational data; an edge-processing layer responsible for filtering, feature extraction, and lightweight inference; a diagnostic layer estimating defect type, location, and severity; a digital twin layer updating the virtual belt state and degradation model; and a decision-support layer generating alarms, maintenance recommendations, and inspection schedules. Such an architecture would enable a transition from reactive damage detection toward predictive and explainable maintenance planning.

### 6.6. Limitations and Practical Challenges

Despite significant advances in diagnostic methods, numerous limitations still hinder practical industrial implementation. The most important challenges include

- limited availability of large annotated training datasets,
- variability of environmental and operational conditions,
- limited interpretability of deep learning models [100,101,102,103],
- transferability issues between different industrial installations,
- lack of standardized evaluation procedures for diagnostic systems.

In addition to environmental robustness and data availability, limited model interpretability remains an important deployment barrier, as discussed in Section 6.5 in the context of Explainable AI.

Further development of diagnostic methods therefore requires not only improvements in classification accuracy but also increased robustness to industrial operating conditions and enhanced interpretability of obtained results.

A further limitation concerns the gap between laboratory performance indicators and engineering deployment metrics. Many conveyor belt diagnostic studies report accuracy, precision, recall, F1-score, mAP, or selected inference-speed measures, but only a limited number evaluate system-level industrial indicators such as end-to-end detection latency, false alarm rate, missed detection rate, cleaning or calibration interval, sensor maintenance cycle, integration cost, and robustness during long-term operation. From an engineering perspective, real-time applicability should be assessed not only by model inference speed, but also by image or signal acquisition time, preprocessing time, communication delay, alarm-generation latency, and the ability to operate continuously under dust, vibration, illumination changes, belt-speed variability, and sensor drift. Therefore, future studies should report both algorithmic metrics and deployment-oriented indicators, including real-time performance, false-alarm control, maintenance requirements, and validation under prolonged industrial operation.

## 7. Critical Analysis and Research Gaps

The preceding sections show that conveyor belt diagnostics has progressed from conventional inspection and signal-processing methods toward advanced sensing, machine learning, deep learning, and integrated diagnostic frameworks. However, the transition from sensor-specific detection methods toward robust, transferable, and industrially scalable monitoring systems remains incomplete. Since the operational and methodological limitations of individual technologies have already been discussed in Section 4, Section 5 and Section 6, this section summarizes the main cross-cutting research gaps in a concise matrix form (Table 6).

The matrix indicates that the main barrier to further progress is not the lack of individual sensing technologies or classification algorithms, but the limited integration of diagnostic information across sensing modalities, operating conditions, and maintenance decision levels. Future conveyor belt diagnostic systems should therefore move from isolated defect-detection algorithms toward integrated diagnostic ecosystems capable of combining heterogeneous sensing data, robust feature representation, explainable artificial intelligence, multimodal fusion, and deployment-oriented performance evaluation.

The limited integration of operational failure modes, such as belt looseness, with physical belt-damage diagnostics also remains an important research gap. Future systems should combine structural belt-condition assessment with operational indicators related to belt tension, speed variation, load distribution, drive response, and current-signal anomalies.

## 8. Future Directions and Emerging Perspectives

The rapid development of diagnostic technologies used for conveyor belt condition monitoring indicates a gradual transition from systems based on single sensing technologies toward more integrated, intelligent, and autonomous diagnostic frameworks. Advances in sensing technologies, data-processing methods, and artificial intelligence algorithms are currently transforming the design philosophy of condition monitoring and fault diagnosis systems. Traditional approaches based mainly on periodic inspections and reactive maintenance are progressively being replaced by solutions employing continuous monitoring, large-scale data analysis, and predictive maintenance strategies [8,9,10,11].

One of the most promising future directions appears to be the integration of data originating from multiple sensing technologies. At present, most diagnostic systems rely on a single data modality and focus on selected manifestations of the degradation process [14,15]. Vision-based systems effectively identify surface defects [16,17,18,46,47,48,49,50], magnetic methods enable assessment of steel cord and internal reinforcement condition [19,20,21,56,57,58,59], whereas ultrasonic techniques provide information related to thickness variations and wear development [22,23,24]. However, each of these approaches captures only a limited aspect of the overall degradation process.

Future diagnostic systems will likely increasingly employ multimodal data fusion strategies enabling simultaneous integration of image data, magnetic signals, geometric measurements, and operational parameters of conveyor systems [71,72]. Combining different sensing technologies may improve robustness against environmental disturbances, increase classification performance, and reduce the risk of incorrect diagnostic interpretation. Particularly important will be the development of systems capable of simultaneously monitoring both the working belt surface and the evolution of internal defects responsible for the loss of load-carrying capacity.

At the same time, rapid progress is also being observed in artificial intelligence methods applied to diagnostic data analysis [29,69]. In recent years, deep-learning-based approaches have become particularly important because they enable automatic extraction of feature representations without the need for manual design of diagnostic parameters [69,80]. Such methods have demonstrated high effectiveness in image-based diagnostics [15,18,33,34,35], however, their industrial deployment is still limited by issues related to training data availability, model interpretability, and transferability between different industrial installations.

In the coming years, increasing attention can be expected toward methods enabling more effective utilization of limited data resources. Particularly promising approaches include transfer learning, self-supervised learning, and models enabling the development of universal diagnostic data representations [69,89]. These methods may become especially important in industrial applications, where the preparation of large manually annotated datasets remains expensive and time-consuming. In this context, unsupervised and one-class anomaly detection methods trained on healthy-condition data are particularly promising because they can support early warning and novelty detection when representative fault examples are unavailable. Another important direction of future development involves moving diagnostic processing closer to the point of data acquisition. Increasing computational capabilities of sensing devices together with the development of lightweight deep learning models have led to growing interest in Edge AI solutions enabling diagnostics directly at the measurement-system level [94]. Such approaches may reduce data transmission latency, improve robustness against communication problems, and enable faster responses to developing damage.

Increasing importance is also being attributed to the interpretability of artificial intelligence methods. Despite high diagnostic performance, deep learning models still frequently operate as black-box systems, making it difficult to clearly explain classification decisions [100,101,102,103]. In industrial applications, explainability is particularly important due to operational safety requirements and the need to build operator trust in monitoring systems. Consequently, further development of Explainable Artificial Intelligence (XAI) methods improving the transparency of diagnostic models can be expected.

Another promising research direction involves integration of diagnostic systems with digital twin technologies [104,105]. Combining monitoring-system data with degradation models and operational simulations may enable more comprehensive evaluation of conveyor belt technical condition. Such solutions may provide the foundation for transitioning from conventional condition monitoring toward predictive maintenance systems capable of damage progression forecasting and maintenance optimization [8,9,10,11].

Perhaps the most important future research direction, however, is the development of more universal diagnostic frameworks enabling integration of data originating from different sensing technologies. As demonstrated throughout this review, most currently developed methods remain strongly dependent on a specific sensing modality, which limits result comparability and the transferability of developed solutions between different industrial environments.

The future development of conveyor belt diagnostics will therefore likely depend not only on further improvements in individual sensing technologies but primarily on the creation of integrated diagnostic ecosystems combining multiple data modalities, advanced information representation methods, and intelligent decision-support systems. The development of such solutions may provide the foundation for more universal, robust, and scalable condition monitoring systems capable of reliable operation under complex industrial conditions.

## 9. Conclusions

Conveyor belts constitute one of the fundamental components of bulk material handling infrastructure, and their reliability directly affects process safety, operational efficiency, and the economic performance of industrial systems. Increasing demands for continuous operation of industrial installations have significantly increased the importance of advanced diagnostic methods enabling early damage detection and continuous condition monitoring of conveyor belts.

This paper presented a comprehensive review of conveyor belt damage detection and analysis methods, with particular emphasis on sensing technologies and diagnostic signal analysis approaches. The main degradation mechanisms of conveyor belts were discussed together with their representation in data acquired from different diagnostic systems. The current state of development of vision-based systems, magnetic techniques, ultrasonic methods, and X-ray imaging was analyzed, highlighting their capabilities, limitations, and areas of application.

The conducted analysis demonstrated that the effectiveness of conveyor belt monitoring systems depends not only on the applied sensing technology but also on data representation methods, signal-processing techniques, and diagnostic feature extraction procedures. The development of machine learning and deep learning methods has significantly improved the possibilities of automatic damage detection and classification; however, most currently developed solutions remain strongly dependent on specific sensing technologies and are typically limited to single data modalities.

The critical literature analysis enabled the identification of several key research challenges limiting the further development of conveyor belt diagnostics. The most important include the lack of unified damage representation across different sensing technologies, limited availability of public benchmark datasets enabling objective comparison of diagnostic methods, insufficient validation under real industrial conditions, and still relatively limited application of multimodal data integration approaches.

Analysis of current research trends indicates that future diagnostic systems will likely increasingly rely on the integration of multiple sensing technologies, artificial intelligence methods, digital twin concepts, and the transition from reactive diagnostics toward predictive maintenance systems. Particularly promising appears to be the development of more universal diagnostic data representation methods enabling integration of information originating from different sensing technologies.

Future progress in conveyor belt diagnostics will therefore depend not only on further advances in individual sensing technologies but primarily on the development of integrated diagnostic ecosystems combining advanced sensing systems, intelligent data-analysis methods, and decision-support tools. Such solutions may provide the foundation for more reliable, scalable, and robust condition monitoring systems supporting the ongoing transformation of industry toward intelligent maintenance and Industry 4.0-oriented infrastructure.

## Figures and Tables

**Table 1:** Diagnostic data characteristics generated by selected sensing technologies.

Sensing Technology	Data Representation	Typical Defect Signature	Typical Processing Methods
Machine vision	RGB/line-scan image, 3D surface data	texture changes, edges, geometric discontinuities	filtering, segmentation, contour analysis, CNN/object detection
MFL	One-dimensional or multichannel magnetic signal	localized peaks, amplitude distortions, spatial anomalies	filtering, baseline correction, lift-off compensation, wavelet/EMD, statistical features
Ultrasound	thickness profile, echo signal	thickness reduction, attenuation, local discontinuities	smoothing, TOF analysis, trend analysis, spectral/time–frequency features
MCSA/current-signal monitoring	motor current signal, harmonic components	load-related anomalies, tracking/slippage-related current changes	FFT, higher-order spectra, bicoherence, tricoherence, CCSM3, statistical thresholds
X-ray	grayscale internal image	reinforcement discontinuity, cord misalignment, splice anomaly	image enhancement, segmentation, object detection, CNN-based classification

**Table 2:** Industrial failure modes and sensitivity degradation mechanisms of selected conveyor belt diagnostic technologies.

Technology	Main Industrial Disturbance or Failure Mode	Effect on Diagnostic Sensitivity	Engineering Mitigation	Remaining Limitation
Machine vision	Dust, surface contamination, variable illumination, shadows, vibration, lens contamination	Reduced image contrast, missed small defects, false alarms caused by stains or background texture	Controlled illumination, shielding, lens cleaning, image enhancement, adaptive thresholding, robust object-detection models	Quantitative degradation of detection accuracy under dust and illumination variability is rarely reported systematically; most studies report performance on laboratory or dataset-based images rather than controlled environmental stress tests
3D/laser vision	Belt vibration, variable belt height, surface reflectance changes, calibration drift	Distorted geometry, unstable point clouds, reduced accuracy of rip-width or surface-profile estimation	Rigid mounting, calibration procedures, geometric filtering, point-cloud clustering, outlier removal	High spatial accuracy requires stable sensor geometry and regular calibration
MFL	Lift-off variation, magnetization instability, sensor-head orientation, belt speed variation, mechanical vibration	Reduced signal amplitude and SNR, shifted peak position, distorted signal shape, increased risk of false positives or false negatives	Stable mechanical guidance, reference calibration, baseline correction, multi-channel sensing, lift-off compensation, sensor-orientation control	Lift-off compensation has engineering limits because increasing sensor-to-belt distance decreases the useful defect signal and may reduce defect-size estimation reliability
Ultrasonic testing	Unstable sensor geometry, poor coupling conditions, surface unevenness, belt vibration, temperature effects	Loss of echo quality, noisy thickness profiles, reduced repeatability of local wear measurements	Fixed transducer arrays, signal averaging, smoothing, calibration on reference samples, stable mounting	Continuous online measurement requires stable geometry and sufficient signal quality during belt motion
MCSA/current-signal monitoring	Load variability, belt looseness/tension variation, drive dynamics, gearbox condition, speed changes, electrical noise	Indirect symptom representation; possible confusion between belt faults and drive/load disturbances	Baseline learning, operating-regime normalization, harmonic analysis, higher-order spectral features, integration with speed/load data	Effective mainly for operational faults such as mistracking, looseness or slippage; limited ability to localize physical belt damage
X-ray imaging	High equipment cost, radiological safety requirements, shielding constraints, speed–resolution trade-off	Limited feasibility of continuous deployment, possible reduction in image quality at high inspection speed	Shielded inspection stations, controlled acquisition geometry, automated image enhancement and defect detection	Industrial use is constrained by safety, cost, and integration complexity rather than defect visibility alone

**Table 3:** Representation of conveyor belt defects in different sensing modalities.

Damage Type	Vision Systems	MFL	Ultrasound	X-Ray
Abrasive wear	Texture modification	–	Thickness reduction	Limited visibility
Cut damage	Geometric discontinuity	–	Local anomaly	Visible
Longitudinal rip	Shape anomaly	–	Local discontinuity	Visible
Delamination	Limited visibility	–	Reflection anomaly	Visible
Steel cord damage	Usually invisible	Magnetic anomaly	Limited sensitivity	Visible

**Table 4:** Mapping between conveyor belt damage types, diagnostic data representations, recommended processing methods, and parameter-selection guidance.

Damage Type	Typical Data Representation	Characteristic Diagnostic Information	Commonly Applied Processing Methods	Parameter-Selection Guidance
Abrasive wear	surface image, thickness profile	texture degradation, thickness loss	GLCM, LBP, trend analysis	Window size should reflect expected wear-texture scale; trend interval should match inspection frequency.
Cut damage	image	geometric discontinuity	edge detection, segmentation	Edge thresholds and morphological filters should suppress belt texture, scratches, and contamination.
Longitudinal rip	image, profile	elongated anomaly	contour analysis, CNN, object detection	Region continuity, aspect ratio, and minimum length should reflect elongated rip geometry.
Steel cord break	MFL signal	localized magnetic peak	FFT, wavelet, EMD, statistical features	Analysis window and wavelet/EMD scale should match belt speed, sampling frequency, and expected anomaly width.
Corrosion	MFL signal	distributed signal distortion	spectral analysis, entropy, wavelet features	Longer windows and baseline correction are recommended because signal changes are distributed and low-amplitude.
Delamination	ultrasonic signal	reflection anomaly	STFT, wavelet transform	STFT/window length or wavelet scale should balance temporal localization and frequency resolution.

**Table 5:** Classical machine learning methods applied in conveyor belt diagnostics.

Method	Typical Conveyor Belt Application	Typical Input	Advantages	Limitations
SVM	MFL defect classification, wear assessment	Statistical features	Good performance with limited data	Feature engineering required
Random Forest	defect classification from handcrafted features	Handcrafted features	Robustness	Reduced interpretability
k-NN	simple wear/cut recognition	Geometric/statistical features	Simplicity	Computational cost
ANN	signal-based anomaly detection	Signal features	Nonlinear modelling	Feature selection required
One-Class SVM	anomaly or novelty detection based on healthy-condition data	normal-condition feature vectors from MFL, current, vibration, ultrasonic, or image descriptors	Does not require labelled fault examples	Limited fault attribution; sensitivity to kernel, threshold, and operating-condition changes

**Table 6:** Critical research gaps and transition requirements in conveyor belt diagnostics.

Research Gap	Current State of the Literature	Consequence for Conveyor Belt Diagnostics	Required Development Direction
Lack of unified damage representation	Damage is represented differently depending on sensing modality, for example, as texture change, geometric discontinuity, magnetic anomaly, thickness loss, or internal structural defect.	Results obtained using different diagnostic systems are difficult to compare, benchmark, and integrate.	Development of common diagnostic descriptors including damage type, location, severity, spatial extent, temporal evolution, data quality, and diagnostic confidence.
Sensor-specific diagnostic pipelines	Most methods are designed for a single input type, such as RGB images, 3D point clouds, MFL signals, ultrasonic profiles, or X-ray images.	High performance can be achieved in selected tasks, but transferability between sensing systems and industrial installations remains limited.	Transition from modality-specific pipelines toward shared feature spaces and diagnostic interpretation layers operating across heterogeneous data sources.
Limited benchmark datasets and fault-label scarcity	Many studies rely on proprietary industrial datasets or laboratory measurements, and labelled fault examples are often scarce or highly imbalanced.	Reproducibility and objective comparison of algorithms remain limited; supervised models may be difficult to train for rare or previously unseen damage states.	Creation of benchmark datasets with standardized damage labels and operating metadata, together with broader use of unsupervised, one-class, and self-supervised anomaly detection methods trained on healthy-condition data.
Insufficient industrial validation	Many methods report laboratory accuracy, precision, recall, F1-score, mAP, FLOPs, or FPS, but only limited long-term industrial validation is available.	High reported algorithmic performance may not translate into reliable operation in real conveyor systems.	Validation under prolonged industrial operation, including dust, vibration, illumination variability, belt-speed changes, lift-off variation, sensor drift, and maintenance constraints.
Limited engineering-performance reporting	Deployment-oriented indicators such as end-to-end latency, false alarm rate, missed detection rate, calibration interval, maintenance cycle, and integration cost are rarely reported systematically.	Practical feasibility, lifecycle cost, and operational reliability of diagnostic systems remain difficult to assess.	Reporting of both algorithmic and engineering metrics, including real-time performance, false-alarm control, maintenance requirements, integration complexity, and long-term stability.
Limited interpretability of AI models	Deep-learning methods often provide high detection performance but limited transparency of decision-making.	Reduced operator trust and difficulties in fault attribution may limit acceptance in industrial maintenance systems.	Integration of Explainable AI tools such as Grad-CAM, saliency maps, SHAP, and LIME to support model verification, fault attribution, and operator-oriented decisions.
Underdeveloped multimodal data fusion	Existing fusion studies are mainly concentrated on selected cases such as infrared–visible or audio–visual tear detection, while full integration of vision, MFL, ultrasound, and operational data remains rare.	Single-modality systems observe only selected manifestations of belt degradation and may be vulnerable to modality-specific disturbances.	Development of multimodal fusion frameworks combining complementary surface, internal, geometric, magnetic, acoustic, and operational information.
Weak connection with predictive maintenance	Many diagnostic systems focus on defect detection or classification rather than degradation modelling, remaining useful life estimation, or maintenance planning.	Diagnostic outputs are not always directly translated into maintenance decisions or long-term asset management strategies.	Integration of sensing data, degradation models, Edge AI, Explainable AI, and Digital Twin frameworks for predictive and decision-oriented maintenance.

## Data Availability

No new data were created or analyzed in this study. Data sharing is not applicable to this article.
